# Supplementary material for: Permittivity-asymmetric qBIC metasurfaces for refractive index sensing
Source: Nanophotonics. 2025 Nov 24;14(27):5311–21. doi: 10.1515/nanoph-2025-0415 (PMC12717935; doi:10.1515/nanoph-2025-0415)
Supplement: Supplementary file 1 — Supplementary Material Details [file j_nanoph-2025-0415_suppl_001.docx]

Permittivity-asymmetric qBIC metasurfaces for refractive index sensing

Supplementary information

Tables of contents

Note 1: Potential application of the metasurfaces with reversibility between RSP-BIC and qBIC.

Figure S1. Schematic overview of nanofabrication for ε-qBIC metasurface.

Figure S2: Centroid wavelength calculation.

Figure S3: Measured transmittance spectra of metasurfaces in the air environment.

Figure S4. Simulation of Q-factor vs intensity modulation response for g-qBIC and ε-qBIC.

Figure S5. RI sensing with a g-qBIC metasurface of initial Q-factor 171 in air.

Figure S6. Mechanism of the ε-qBIC.

**Note 1: Potential application of the metasurfaces with reversibility between RSP-BIC and qBIC.**

The reversibility between RSP-BIC and qBIC is essentially achieved by tuning γ_rad_, which controls the coupling of the resonance to the far field. Compared with the conventional approach of damping resonances through γ_int_, this mechanism allows direct on/off switching of resonances under conditions of strong field enhancement. Based on this control scheme, several potential applications can be considered.

**On/Off Switchable Filters**. By tuning γ_rad_, the mode can be either fully decoupled from the far field (γ_rad_ ≈ 0, resonance-free state) or strongly coupled (γ_rad_ > 0, resonant state). This enables an optical filter with a clear on/off behavior: in the “off” state, the system remains transparent with minimal crosstalk and absorption, while in the “on” state, a strong resonance appears. Such a capability is useful for active optical filtering in photonic circuits.

**Simple and practical sensing devices**. Permittivity-asymmetry-induced RSP-BIC represents a non-stationary state with respect to the surrounding refractive index. Any change in the environment can induce a transition between RSP-BIC and qBIC, which can be detected as a clear switching of the resonance. When the refractive index of the environment is varied, the permittivity asymmetry between the two rods in the unit cell is modified, directly affecting γ_rad_. As a result, the system undergoes a transition from a transparent state (RSP-BIC, γ_rad_ ≈ 0, no coupling to the far field) to a resonant state (qBIC, γ_rad_ > 0), leading to a distinct spectral on/off contrast. In the visible regime, for example, the sensor appears transparent in the RSP-BIC regime, while a noticeable color change occurs once the environment drives the system into the qBIC regime. Since this transition is governed by radiative coupling rather than intrinsic loss (γ_int_), the contrast is stronger than conventional resonance shifts, while still maintaining high Q-factors and strong near-field enhancement.


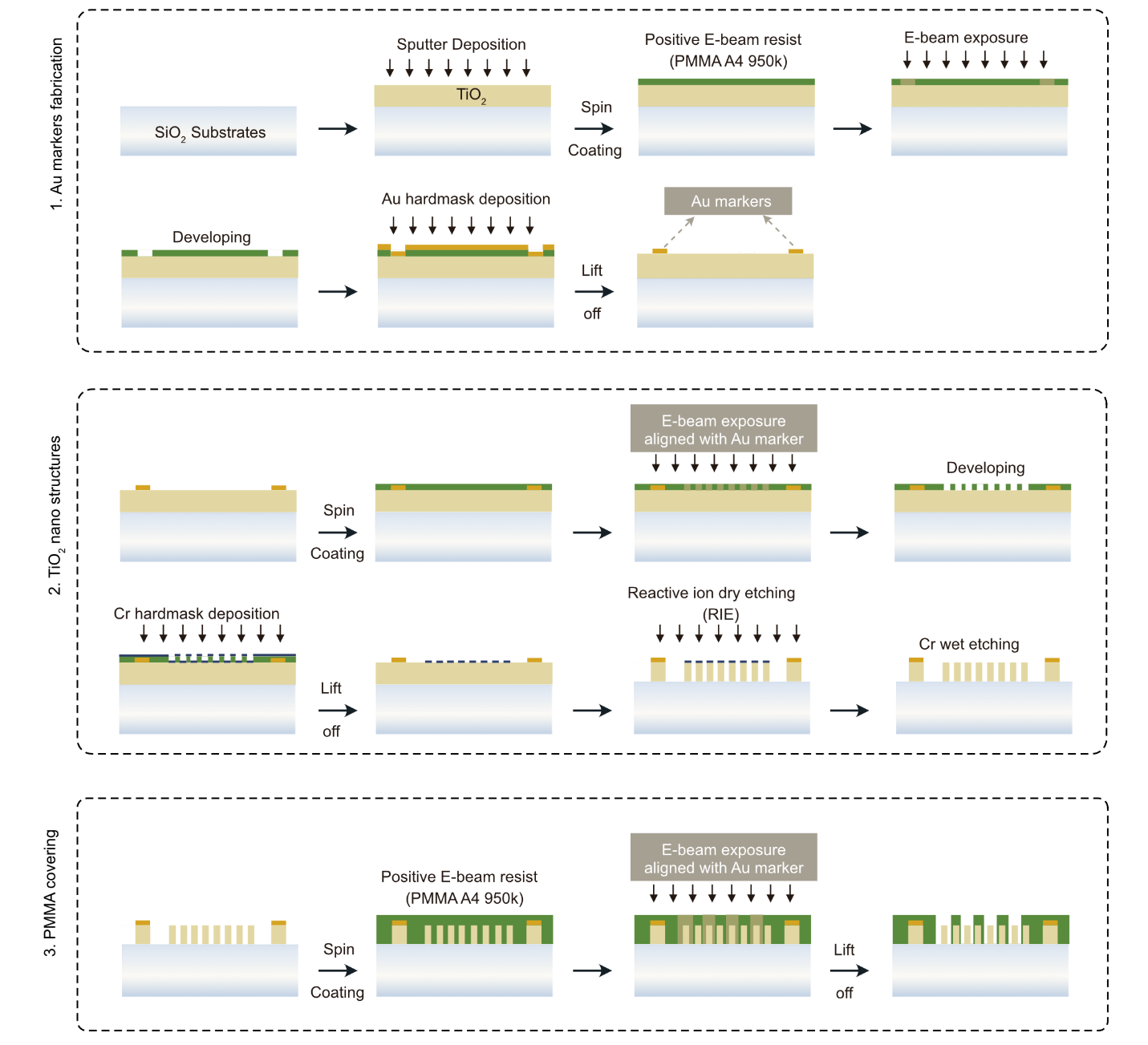


**Figure S1. Schematic overview of nanofabrication for ε-*q*BIC metasurface.** It includes three main steps, the fabrication of Au marker systems, TiO_2_ nanostructures, and selective patterning on PMMA.


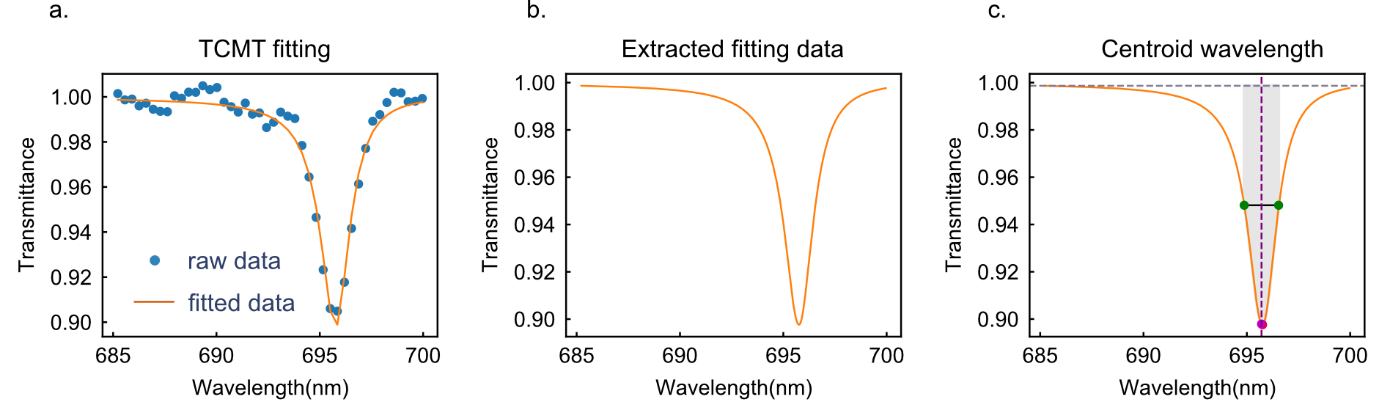


**Figure S2.** **Centroid wavelength calculation.** a. Raw discrete transmittance data fitted using the temporal coupling model theory (TCMT) to fit for the raw discrete spectral data. b. Clean fitted curve extracted from the TCMT model, applied for subsequent analysis. c. Illustration of centroid wavelength extraction from the fitted spectrum. The shaded gray area represents the integration region, and the vertical dashed line marks the calculated center of mass. This method follows established procedures reported in prior studies.


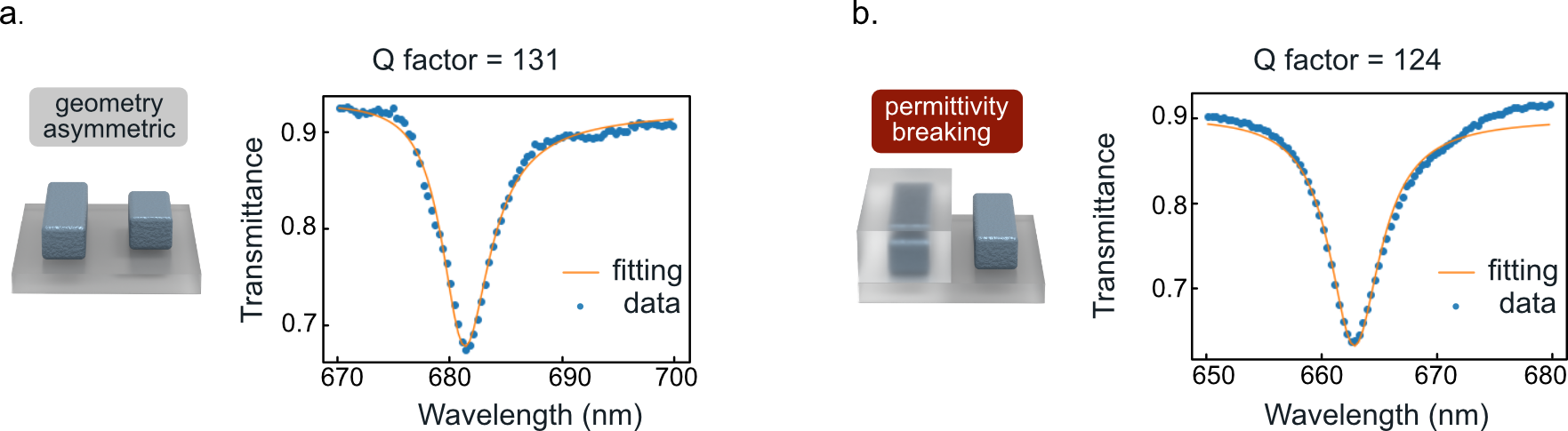


**Figure S3. Measured transmittance spectra of metasurfaces in the air environment.** a. The transmittance spectra of g-*q*BIC metasurface with Q factor 131. b. The transmittance spectra of ε-*q*BIC metasurface with Q factor of 124.


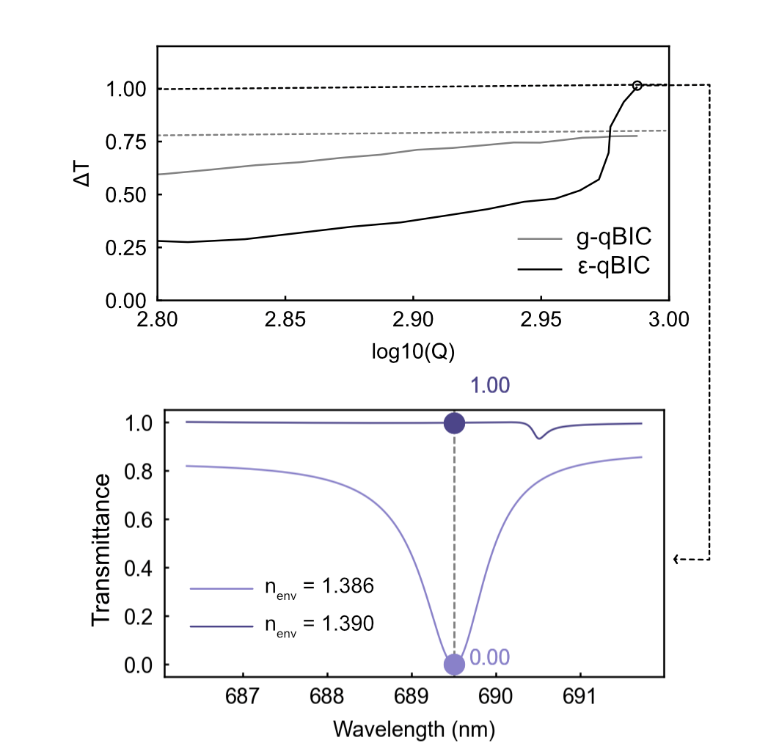


**Figure S4. Simulation of Q-factor vs intensity modulation response for g-qBIC and ε-qBIC.**

Simulations were performed to analyze the sensitivity performance of g-qBIC and ε-qBIC structures by sweeping the geometric asymmetry in g-qBIC and the cladding refractive index (n_cov_) in ε-qBIC. For each configuration, the Q-factor was extracted from the simulated transmittance spectra. Subsequently, the environmental refractive index was varied from 1.386 to 1.390 (Δn = 0.004) to evaluate the corresponding intensity modulation signal ΔT at a fixed wavelength. The results show that in ε-qBIC, when n_cov_ is tuned to closely match the surrounding environment, the system enters a high-Q regime where even a small RI change can strongly affect the radiative loss channel. When n_cov_ equals the environmental index, the qBIC resonance can be even restored into a BIC. This leads to an abrupt transition from a resonant to a non-resonant state, producing a unity modulation depth. In contrast, the ΔT signal in g-qBIC saturates at approximately 0.77.


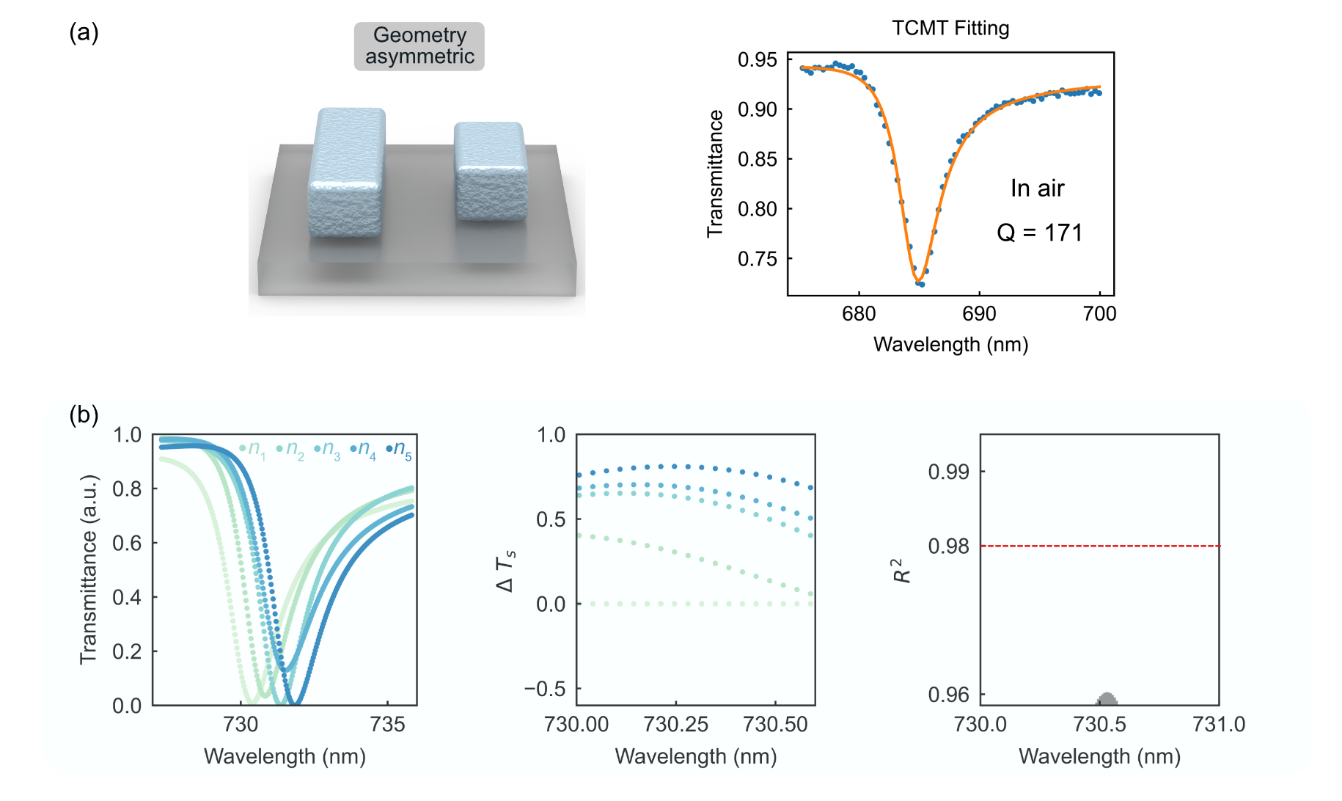


**Figure S5. RI sensing with a g-qBIC metasurface of initial Q-factor 171 in air.** (a) Left: schematic of the geometry-broken metasurface. Right: experimental spectrum in air with TCMT fitting, yielding a Q-factor of 171. (b) Left: transmission spectra measured under different refractive indices (n₁ to n₅, ranging from 1.386 to 1.402 with a step size of Δn = 0.004). Middle: spectral differences relative to the first spectrum, processed in the same way as in the main text. Right: R² values obtained from linear fitting across multiple wavelengths, used to evaluate the linearity of the single-wavelength intensity variation response.

This additional experiment was conducted to further examine the spectra fluctuations in Fig. 4b. While the spectra in Fig. 4b exhibited noticeable fluctuations that may originate from disturbances in the measurement process (e.g., light source intensity variations), the spectra here show a relatively more stable response. Nevertheless, after applying the same data processing procedure, single-wavelength differential analysis followed by linear fitting, the R² distribution still reaches only about 0.96 at maximum, consistent with the result of g-qBIC in Fig. 4d of the main text and well below 0.98. This confirms that the limited R² values are mainly related to the intrinsic behavior of the g-qBIC response under single-wavelength differential sensing.

**
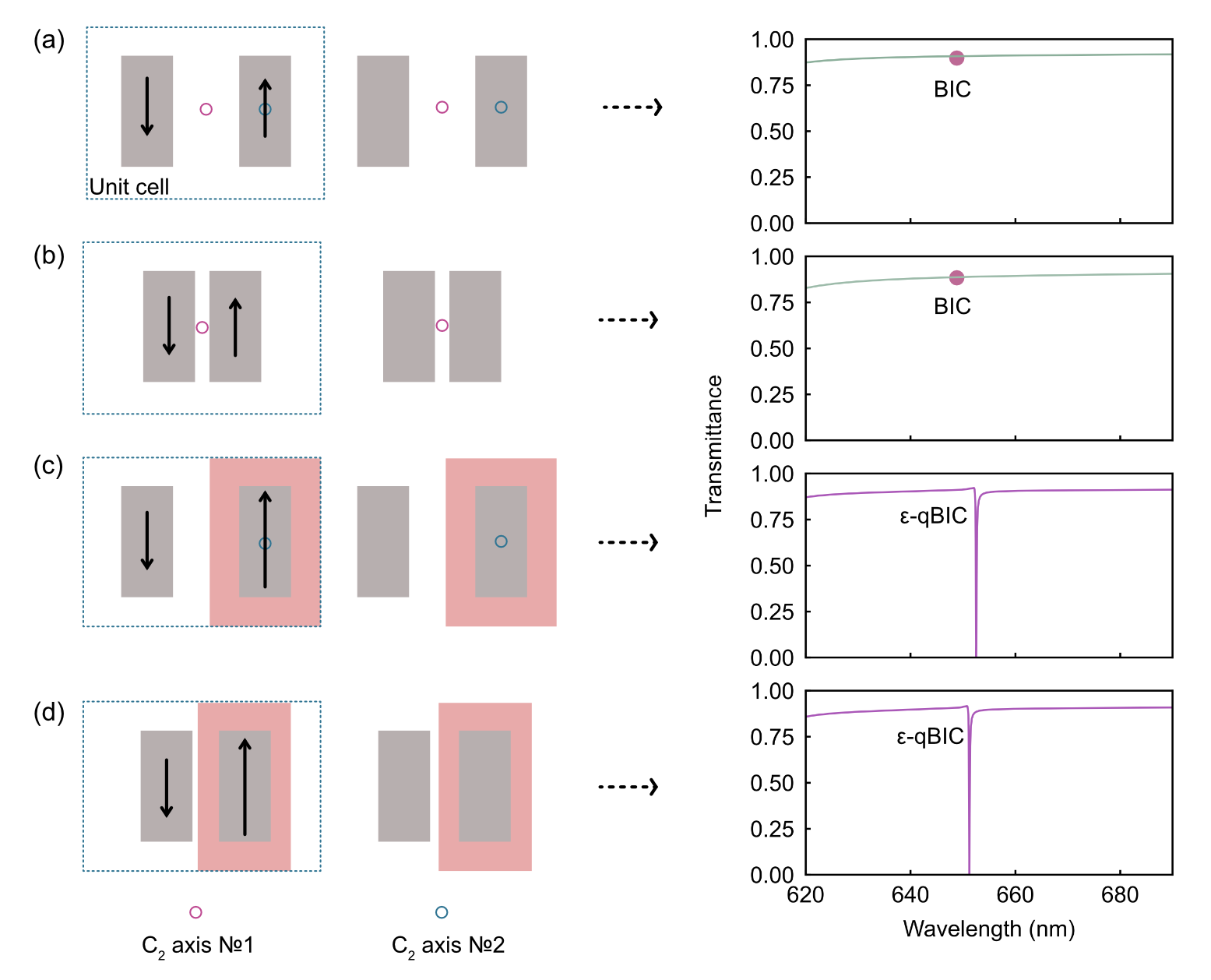
**

**Figure S6. Mechanism of the ε-qBIC.** (a) Metasurface from the main text with equally spaced nanorods exhibiting a symmetry-protected antiparallel dipole BIC. (b) Shifting each rod by 10 nm each doubles the period and breaks the C₂ axis №2, while the BIC at the Γ-point remains preserved. (c) Introducing a 20 nm-thick PMMA layer to the structure with equally spaced nanorods breaks the C₂ axis №1 and transforms the symmetry-protected BIC into an ε-qBIC, as indicated by the transmittance spectrum. (d) The same transition from BIC to ε-qBIC is observed for the structure with shifted nanorods lacking both C₂ axes.

We interpret $\varepsilon$-qBIC as a resonance originating from a symmetry-protected BIC. The original mode is an antiparallel dipole resonance and can be described by a pair of nanorods (Fig. S6(a)), where the corresponding structure possesses multiple C_2_ rotational symmetries. We focus on the two axes marked as red (axis №1) and blue circle (axis №2) in Fig. S6.

By shifting the nanorods we double the period, break the C_2_ axis №2 and introduce Brillion zone folding (BZF). However we still cannot access the mode at the Г-point (Fig. S6(b)), as it remains protected by the remaining C_2_ rotational symmetry described by the axis №1.

In our work it is crucial to break exactly this particular rotational symmetry, which we achieve by introducing a PMMA coverage. The mechanism responsible for transforming symmetry-protected antiparallel dipole BIC into a qBIC does not depend on whether the rotational symmetry associated with C_2_ axis №2 is preserved (Fig. S6(c)) or broken (Fig. S6(d)).

However, the mechanism for the metasurface shown in Fig. S6(c) can also be interpreted using the BZF framework [1], [2], since introducing the PMMA coating both breaks the necessary C₂ axis №1 and doubles the period.

References

[1] W. Wang, Y. K. Srivastava, T. C. Tan, Z. Wang, and R. Singh, “Brillouin zone folding driven bound states in the continuum,” *Nat Commun*, vol. 14, no. 1, p. 2811, 2023, doi: 10.1038/s41467-023-38367-y.

[2] K. Sun *et al.*, “Infinite-Q guided modes radiate in the continuum,” *Phys Rev B*, vol. 107, no. 11, p. 115415, Mar. 2023, doi: 10.1103/PhysRevB.107.115415.
